# The Keith Edward scoring system: A case control study

**DOI:** 10.4103/0970-2113.48894

**Published:** 2009

**Authors:** Supriya Sarkar, Dilip Kumar Paul, Sudipta Chakrabarti, Nirmal Kumar Mandal, A.G. Ghoshal

**Affiliations:** *Department of Chest Medicine, Nil Ratan Sircar Medical College, Kolkata, India*; 1 *Department of Pediatric Medicine, Nil Ratan Sircar Medical College, Kolkata, India*; 2 *Department of Pathology, Nil Ratan Sircar Medical College, Kolkata, India*; 3 *Department of Community Medicine, BSMC, Bankura, India*; 4 *Department of Chest Medicine, Nil Ratan Sircar Medical College, Kolkata, India*

**Keywords:** Childhood tuberculosis, diagnosis, scoring system

## Abstract

**Objective:**

The World health organization (WHO) has accepted Keith Edward scoring system for the diagnosis of childhood tuberculosis (TB). In the present study, we evaluated this scoring system.

**Methods and Results:**

We included 53 children with confirmed TB involving different organs, admitted in NB Medical College, during two years period as cases; and 50 randomly selected, age, sex, and organ matched confirmed non-TB cases as controls. We noticed 15.1% false negative and 22% false positive results in our study, and the scoring system had 84.9% sensitivity, 78% specificity, and 80.36% positive predictive value. Likelihood ratio positive (LR+) was 3.86, likelihood ratio negative (LR–) was 0.19, and overall agreement was 81.55%. We observed that Keith Edward scoring system was less effective in children suffering from non-TB chronic diseases (false positive rate: 45.5%). We found no significant difference in nutritional status between study and control groups (*P* = 0.65). We noticed that more than 15-mm indurations for tuberculin test were specific for TB in children.

**Conclusion:**

We concluded that Keith Edward scoring system is good for public health purpose, but there is a scope for improvement, and further study is required for this purpose.

## INTRODUCTION

Tuberculosis (TB) is familiar to human being since prehistoric ages. The diagnosis of TB still remains elusive, particularly in childhood TB. Childhood TB now constitutes 40% of the total burden.^1^ In the year 2000 alone, an estimated 8.3 million new cases of TB occurred in India, of which 884,019 cases occurred in children.^2^ It is being increasingly realized that management of childhood TB is crucial as infection acquired during childhood reactivates to adult disease, which in turn, maintains the chain of transmissions. Cornerstone of the problem of diagnosis of childhood TB is the absence of confirmatory tests, like sputum microscopy, etc. Spurts of literature, both from developed and developing countries, focus categorically on the issue of diagnostic dilemma of childhood TB. Different scoring systems were developed combining clinical presentation, tuberculin test, chest X-ray, tissue histopathology, and even antitubercular drug trial. Although helpful, none of them proved to be satisfactory and flawless. There are scoring systems proposed by Stegen *et al*,^3^ Nair and Philip^4^, etc. Scoring system proposed by Dr. Keith Edwards [Table 1] was endorsed and advocated by WHO^5^ for use in National TB Control Programs of different countries. Our Revised National TB Control Program has incorporated this scoring system. In this case control study, we have evaluated the Keith Edwards scoring system.

**Table 1:** Scoring system adopted in this study

Features	Score				
	
	0	1	2	3	4
Length of illness (weeks)	<2	2–4	-	>4	-
Nutrition (weight for age; %)	>80	60–80	-	<60	-
Family history of tuberculosis	None	Reported	-	Proven	-
Tuberculin test	-	-	-	Positive	-
Painless lymphadenopathy with or without sinus	-	-	-	Positive	-
Unexplained fever not responding to antimalarial drugs	-	-	Positive	-	-
Malnutrition not improved after four weeks	-	-	-	Positive	-
Angle deformity of spine	-	-	-	-	Positive
Joint/bone swelling, sinuses	-	-	-	Positive	-
Unexplained abdominal mass or ascites	-	-	-	Positive	-
CNS: changes in temperament, fits, or coma	-	-	-	Positive	-

## MATERIALS AND METHODS

A hospital-based case control study was conducted in the Departments of Respiratory Medicine and Pediatric Medicine of North Bengal Medical College and Hospital (NBMCH), Darjeeling District, West Bengal. We excluded infants and children above 12 years of age (as they can expectorate and sputum can be examined). The period of study was from January 2001 to December 2002. All 55 children with confirmed TB admitted during the period of study at NBMCH were taken as cases in the study. Children with more than three weeks of respiratory symptoms, with chest X-ray (CXR) findings suggestive of TB, and persistence of CXR findings after 10 days of antibiotic therapy were diagnosed with pulmonary TB. Lymphocytic exudative fluid in serous cavity with adenosine deaminase level >40 Unit/L was considered as TB of that particular serous cavity (pleural, pericardial, meningeal, or ascities). TB lymphadenopathy was confirmed by FNAC, and demonstration of acid fast bacilli (AFB). Miliary TB was diagnosed by typical clinical picture, miliary shadow in CXR, and absence of peripheral blood eosinophilia. Dissiminated TB was diagnosed when diseases involved more than two noncontiguous organs, and TB was confirmed in at least one organ. Moreover for further confirmation, all cases recieved a therapeutic trial with antitubercular drugs. As two children failed to respond to clinical trial, we excluded them from the study. Ultimately, 53 children were taken as study cases. Fifty confirmed non-TB cases were selected randomly as age (*P* = 0.27) and sex (*P* = 0.27) matched controls [Table 2]. Controls were selected to match the types of cases. Cases of lymphomas were taken as control for TB lymph nodes; malnutrition and chronic diarrhea for miliary and disseminated TB; pneumonia, chronic cough due to asthma, and bronchiolitis for pulmonary TB; rheumatic carditis for TB pericardial effusion; pyogenic meningitis for TB meningitis; Indian childhood cirrhosis, gastroenteritis, and nephritic syndrome for abdominal TB. We excluded known HIV-positive children or children of HIV-positive mothers. However, we did not perform HIV-screening routinely.

**Table 2:** Age and sex distribution in study and control groups

Attributes	Study group no. (%)	Control group no. (%)
Sex		
Males	25 (47.2)	29 (58.0)
Females	28 (52.8)	21 (42.0)
Age (years)		
1–5	14 (26.4)	18 (36.0)
5–10	27 (50.9)	21 (42.0)
10–15	12 (22.6)	11 (22.0)

An independent expert, who was blind to the diagnosis, scored all cases and controls by the Keith Edward scoring system.^5^ Mantoux test was done with 5 TU PPD given intradermally, and it was taken as positive if indurations were ≥10 mm. A total score of ≥7 was taken as positive and a score of <7 was taken as negative for TB.^5^ Treatment of patients was not modified or changed irrespective of the scoring results.

Thereafter, an independent statistician calculated the sensitivity, specificity, positive predictive value, and likelihood ratios of the scoring system by standard statistical methods.

## RESULTS

Twenty pulmonary TB, eight miliary TB, four disseminated TB, two TB pleural effusion, two TB pericardial effusion, four abdominal TB, six TB meningitis, and seven TB lymphadenopathy patients were taken as cases. Fifty age, sex, and disease matched confirmed non-TB cases were taken as controls.

Duration of illness [Table 3] was less than two weeks in five (9.43%) cases, between 2–4 weeks in 22 (41.51%) cases, and above four weeks in 26 (49.06%) of cases; corresponding figures in controls were 36, 42, and 22%, respectively. Duration of illness was found to be significantly longer in the case group (*P* = 0.0001). Nutritional status (percentage of weight for age) was below 60% in 20 (37.74%) cases, between 60–80% in 18 (33.96%) cases, and above 80% in 15 (28.30%) cases; corresponding figures in controls were 22, 42, and 36%, respectively. The case and control groups did not show statistically significant difference (*P* = 0.65) in nutritional status. Family history of TB was found to be significantly high in the study population (35.85% in cases and 18% in controls; *P* = 0.04). Mantoux test results were positive in 75.47% of cases and 24.53% of controls, and the difference was statistically highly significant (*P* = 0.0000002). The Mantoux test results between 15–20 mm were recorded in nine (17.1%) cases and >20 mm in three (5.7%) cases, and significantly none of the controls had indurations >15 mm. Unexplained fever (not responding to antimalarial drugs) was found in 16 (30.2%) cases and 17 (34%) controls. Nutrition not corrected with treatment was found in nine (17%) of cases and three (6%) of controls.

**Table 3:** Comparison of some important parameters of the scoring system

Parameter	Criteria	Study group (%)	Control group (%)	Statistical significance
Duration of illness (weeks)	<2	5 (9.43)	18 (36)	X^2^ = 18.41
	2–4	22 (41.51)	21 (42)	df = 2
	>4	26 (49.06)	11 (22)	*P* = 0.0001
Nutrition (weight for age; %)	<60	20 (37.74)	11 (22)	X^2^ = 0.86
	60–80	18 (33.96)	21 (42)	df = 2
	>80	15 (28.30)	18 (36)	*P* = 0.65
Family history of tuberculosis	Present	19 (35.85)	9 (18)	X^2^ = 4.14,
	Absent	34 (64.15)	41 (82)	df = 1,
				*P* = 0.04
Mantoux test	Positive	40 (75.47)	12 (24)	X^2^ = 27.27,
	Negative	13 (24.53)	38 (76)	df = 1,
				*P* = 0.0000002

In our study, eight cases (15.09%) scored <7: false negative [Table 4]; and 11 (22%) controls scored ≥7: false positive. The calculated sensitivity, specificity, and positive prediction value of the scoring system were 84.9, 78, and 80.36%, respectively. Overall agreement was found to be 81.55%. Likelihood ratio positive (LR+) and likelihood ratio negative (LR−) were 3.86 and 0.19, respectively. The ratio of LR positive to LR negative was 20. The ratio was not as large as desirable (desirable number being 50 or more).^6^

**Table 4:** Validity of scoring system

Scoring	Study group (%)	Control group (%)	Validity
Positive (≥7)	45 (84.91)	11 (22)	Sensitivity = 84.9%
			Specificity = 78%
			Positive predictive
			value = 80.36%
Negative (< 7)	8 (15.09)	39 (78)	LR(+) = 3.86
			LR(−) = 0.19
			Overall agreement
			= 81.55%

## DISCUSSION

Five clinical criteria were thought to be most relevant as predictors of TB in children.^7^ The criteria were: history of contact with a case of TB, positive PPD skin test, persistent cough, low weight for age, and unexplained/prolonged fever. An optimal cut-off point at which TB would be suspected should have a reasonably good predictive value (60–77%).^7^ The predictive value depends on prevalence of disease in the population. In low TB prevalence setting, heavy reliance is placed on a history of contact with household cases of TB and on a positive skin test. For high prevalence setting, more or less equal weightage is assigned to all five elements. In high prevalent areas, contact history and skin tests are less important, and low body weight, prolonged fever, and cough might be more important indicators for TB.^7^ In Keith Edwards scoring system cough has not been taken as criteria for scoring. Moreover, the sensitivity of tuberculin skin test fell significantly in children younger than three years (51%), with HIV coinfection (36%), and with malnutrition (44%).^8^

In our study, we found high false positive error rate (22%) and false negative error rate (15.09%), and the sensitivity, specificity, and positive prediction value of the scoring system were 84.9, 78, and 80.36%, respectively [Table 4]. A study in Pondicherry, India, showed the sensitivity and specificity of the Keith Edward scoring system as 91% and 88%, respectively.^9^ Low sensitivity of the scoring system was due to low scoring in TB pleural/pericardial effusion, TB lymphadenopathy, and early phase of pulmonary TB. Causes of low scoring in those cases might be attributable to the absence of malnutrition in early stage of disease. Family history of smear positive TB was found in only one case. On the other hand, low specificity was due to high scoring in chronic diarrhea and malnutrition. Causes of the high scoring in those controls might be attributable to the presence of malnutrition (3 points) and malnutrition not corrected with treatment (3 points). Absence of statistically significant difference of nutritional status between case and control group (*P* = 0.65) in our study was suggestive of this hypothesis. We also noticed Keith Edward scoring system was less effective for chronic non-TB diseases (more than six weeks duration) where false positive results were high (45.5%).

In conclusion, the Keith Edwards scoring system is effective in the diagnosis of childhood TB, particularly in field conditions. However, it might be less effective in chronic conditions where chances of false positivity are high. Further study on the scoring system might be helpful.
